# Catastrophic household expenditure on health in Nepal: a cross-sectional survey

**DOI:** 10.2471/BLT.13.126615

**Published:** 2014-08-20

**Authors:** Eiko Saito, Stuart Gilmour, Md Mizanur Rahman, Ghan Shyam Gautam, Pradeep Krishna Shrestha, Kenji Shibuya

**Affiliations:** a Graduate School of Medicine, The University of Tokyo, 7-3-1 Hongo, Bunkyo-ku, Tokyo, Japan.; b Deutsche Gesellschaft für Internationale Zusammenarbeit GmbH, Kathmandu, Nepal.; c Institute of Medicine, Tribhuvan University Teaching Hospital, Kathmandu, Nepal.

## Abstract

**Objective:**

To determine the incidence of – and illnesses commonly associated with – catastrophic household expenditure on health in Nepal.

**Methods:**

We did a cross-sectional population-based survey in five municipalities of Kathmandu Valley between November 2011 and January 2012. For each household surveyed, out-of-pocket spending on health in the previous 30 days that exceeded 10% of the household’s total expenditure over the same period was considered to be catastrophic. We estimated the incidence and intensity of catastrophic health expenditure. We identified the illnesses most commonly associated with such expenditure using a Poisson regression model and assessed the distribution of expenditure by economic quintile of households using the concentration index.

**Findings:**

Overall, 284 of the 1997 households studied in Kathmandu, i.e. 13.8% after adjustment by sampling weight, reported catastrophic health expenditure in the 30 days before the survey. After adjusting for confounders, this expenditure was found to be associated with injuries, particularly those resulting from road traffic accidents. Catastrophic expenditure by households in the poorest quintile were associated with at least one episode of diabetes, asthma or heart disease.

**Conclusion:**

In an urban area of Nepal, catastrophic household expenditure on health was mostly associated with injuries and noncommunicable diseases such as diabetes and asthma. Throughout Nepal, interventions for the control and management of noncommunicable diseases and the prevention of road traffic accidents should be promoted. A phased introduction of health insurance should also reduce the incidence of catastrophic household expenditure.

## Résumé

**Objectif:**

Déterminer l'incidence de dépenses catastrophiques de santé des ménages – et les maladies généralement associées à ces dépenses – au Népal.

**Méthodes:**

Nous avons mené une enquête transversale sur la population dans cinq municipalités de la Vallée de Katmandu entre novembre 2011 et janvier 2012. Pour chaque ménage étudié, les dépenses de santé qui sont restées à la charge du ménage dans les 30 jours précédents et qui ont dépassé 10% des dépenses totales du ménage au cours de la même période, ont été considérées comme étant catastrophiques. Nous avons estimé l'incidence et l'intensité des dépenses catastrophiques de santé. Nous avons identifié les maladies les plus généralement associées avec de telles dépenses en utilisant un modèle de régression de Poisson et évalué la distribution des dépenses par quintile économique des ménages en utilisant l'indice de concentration.

**Résultats:**

Dans l'ensemble, 284 des 1 997 ménages étudiés à Katmandu, c.-à- d. 13,8% après correction par pondération de l'échantillonnage, ont signalé des dépenses catastrophiques de santé dans les 30 jours qui ont précédé l'enquête. Après ajustement pour les variables confusionnelles, nous avons pu montrer que ces dépenses étaient associées à des blessures, en particulier celles causées par les accidents de la route. Les dépenses catastrophiques des ménages faisant partie du quintile le plus pauvre étaient associées à au moins un épisode de diabète, d'asthme ou de maladie cardiaque.

**Conclusion:**

Dans une zone urbaine du Népal, les dépenses catastrophiques de santé des ménages furent principalement associées à des blessures et à des maladies non transmissibles comme le diabète ou l'asthme. À travers tout le Népal, des interventions pour le contrôle et la gestion des maladies non transmissibles et pour la prévention des accidents de la route devraient être encouragées. Une introduction progressive de l'assurance maladie devrait également réduire l'incidence des dépenses catastrophiques des ménages.

## Resumen

**Objetivo:**

Determinar la incidencia del gasto catastrófico por motivos de salud de los hogares y las enfermedades generalmente asociadas con dichos gastos en Nepal.

**Métodos:**

Se llevó a cabo una encuesta transversal de la población en cinco municipios del Valle de Katmandú entre noviembre de 2011 y enero de 2012. Para cada hogar encuestado, se consideró catastrófico cualquier gasto de desembolso directo por motivos de salud en los últimos 30 días que hubiera excedido el 10 % del gasto total del hogar durante el mismo periodo. Se estimó la incidencia y el grado de los gastos catastróficos por motivos de salud. Se identificaron las enfermedades asociadas con mayor frecuencia con dichos gastos mediante un modelo de regresión de Poisson y se evaluó la distribución del gasto por quintil económico de los hogares mediante el índice de concentración.

**Resultados:**

En total, se descbrió que 284 de los 1997 hogares estudiados en Katmandú, es decir, un 13,8 % tras el ajuste mediante el muestreo de peso, tuvieron que hacer frente a gastos catastróficos por motivos de salud en los 30 días anteriores a la encuesta. Después del ajuste por factores de confusión, se halló que dicho gasto estaba asociado a lesiones, sobre todo aquellas derivadas de accidentes de tráfico y, en los hogares pertenecientes al quintil más pobre, con al menos un episodio de diabetes, asma o enfermedades cardíacas.

**Conclusión:**

En un área urbana de Nepal, el gasto catastrófico de los hogares por motivos de salud estuvo en su mayoría asociado a lesiones y a enfermedades no transmisibles como la diabetes y el asma. Es necesario fomentar las intervenciones para el control y el manejo de las enfermedades no transmisibles, así como la prevención de los accidentes de tráfico en todo Nepal. La introducción gradual de un seguro médico también podría reducir la incidencia de los gastos catastróficos de los hogares.

## ملخص

**الغرض:**

تحديد معدل حدوث الإنفاق الأسري الكارثي على الصحة في نيبال - والاعتلالات الشائعة المرتبطة بهذا الإنفاق - في نيبال.

**الطريقة:**

قمنا بإجراء دراسة استقصائية سكانية متعددة القطاعات في خمس بلديات في كاتماندو فالي في الفترة من تشرين الثاني/ نوفمبر 2011 إلى كانون الثاني/ يناير 2012. وقد اعتبر الإنفاق من المال الخاص على الصحة خلال الثلاثين يوماً السابقة الذي تجاوز 10% من مجموع الإنفاق الأسري على مدار الفترة ذاتها كارثياً، لكل أسرة خضعت للدراسة الاستقصائية. وقمنا بتقدير معدل حدوث الإنفاق الصحي الكارثي وكثافته. وقمنا بتحديد الاعتلالات المرتبطة على نحو أكثر شيوعاً بهذا الإنفاق باستخدام نموذج ارتداد بواسون، وتقييم توزيع الإنفاق عن طريق الشرائح الخمسية الاقتصادية للأسر باستخدام مؤشر التركيز.

**النتائج:**

بشكل عام، أبلغت 284 من أصل 1997 أسرة خضعت للدراسة في كاتماندو، أي 13.8 % بعد التصحيح عن طريق أخذ عينات الوزن عن إنفاق صحي كارثي خلال الثلاثين يوماً السابقة للدراسة الاستقصائية. وتبين ارتباط هذا الإنفاق، بعد تصحيحه لأغراض تحديد العوامل المؤثرة، بالإصابات لا سيما تلك الناجمة عن حوادث المرور. وكان الإنفاق الكارثي بواسطة الأسر في أفقر شريحة خمسية مرتبطة بنوبة واحدة على الأقل من السكري أو الربو أو مرض القلب.

**الاستنتاج:**

ارتبط الإنفاق الأسري الكارثي على الصحة في إحدى المناطق الحضرية في نيبال في معظمه بالإصابات والأمراض غير السارية مثل السكري والربو. وينبغي تعزيز التدخلات في جميع أرجاء نيبال من أجل مكافحة الأمراض غير السارية وتدبيرها العلاجي وتوقي حوادث المرور. وينبغي أن يؤدي الاستخدام التدريجي للتأمين الصحي أيضاً إلى تقليل معدل حدوث الإنفاق الأسري الكارثي.

## 摘要

**目的:**

确定尼泊尔灾难性卫生家庭支出发生率以及通常与这些支出相关的疾病。

**方法:**

在2011年11月和2012年1月之间，我们在加德满都谷地五个自治市展开基于人口的横断面口调查。对于每个受调查的家庭，在调查前30天预算外卫生支出超过同一时期家庭总开支10%的支出被视为灾难性支出。我们估计灾难性卫生支出发生率和强度。我们使用泊松回归模型识别与此类支出最常相关的疾病，并通过集中指数按照家庭经济五分位数评估支出的分配。

**结果:**

总体来看，加德满都1997户受研究的家庭有284户（即抽样权重调整后13.8%的家庭）报告在调查前30天有灾难性卫生支出。调整混杂因素后，发现这部分支出与损伤有关，特别是道路交通事故引起的伤害。10%最贫穷的家庭中，其灾难性支出与糖尿病、哮喘或心脏病当中至少一种疾病的发病期有关。

**结论:**

在尼泊尔市区，家庭灾难性卫生支出主要与损伤和非传染性疾病相关，如糖尿病和哮喘。在尼泊尔全国，应该提升控制和管理非传染性疾病和预防道路交通事故的干预措施。分阶段引入医疗保险也将降低灾难性家庭支出的发生率。

## Резюме

**Цель:**

Определить влияние катастрофических расходов на медицинские услуги в Непале и выявить, какие заболевания в большинстве случаев связаны с этими расходами, а также частоту возникновения этих заболеваний.

**Методы:**

С ноября 2011 г. по январь 2012 г было проведено перекрестное исследование среди населения пяти муниципальных образований Долины Катманду. Расходы на медицинские услуги для всех домашних хозяйств, принимавших участие в исследовании, признавались катастрофическими, если за предыдущие 30 дней они превышали 10% от общих расходов домашнего хозяйства за этот период.. Были оценены влияние и величина катастрофических расходов на медицинские услуги. Были определены заболевания, которые чаще всего связаны с такими расходами, при помощи модели пуассоновской регрессии и оценено распределение расходов по экономическим квинтилям домашних хозяйств при помощи индекса концентрации.

**Результаты:**

Всего 284 из 1997 домашних хозяйств в Катманду, участвовавших в исследовании, что составляет 13,8% после поправки на размер выборки, сообщили о катастрофических расходах на медицинские услуги за 30 дней, предшествовавших опросу. После поправки с учетом возможных неизвестных факторов эти расходы оказались связаны с травмами, в особенности полученными в результате дорожных происшествий. В домашних хозяйствах, относящихся к самой бедной части населения, были отмечены как минимум по одному случаю диабета, астмы или сердечно-сосудистых заболеваний.

**Вывод:**

В городских районах Непала катастрофические расходы домашних хозяйств на медицинские услуги преимущественно связаны с травмами и неинфекционными заболеваниями, такими как диабет и астма. На всей территории Непала должны быть предприняты оперативные меры по контролю и профилактике неинфекционных заболеваний и предотвращению дорожно-транспортных происшествий. Поэтапное внедрение медицинского страхования должно снизить численность катастрофических расходов домашних хозяйств на медицинские услуги.

## Introduction

In many developing countries, a large proportion of the money spent on health care comes from the out-of-pocket expenditure of patients or their families. In Bangladesh, India and Nepal, for example, this proportion has been estimated to be 48–69%.^1^ Households in such countries can experience financial hardship and often impoverishment as a result of their spending on health care.^2^^–^^5^ In the long term, financial protection against the risk of catastrophic health expenditure at household level can be achieved through tax-based health financing systems or social health insurance schemes – or a combination of both.^6^ In developing countries that have inadequate public funds for health, some transitional measures such as voluntary community-based health insurance schemes may be introduced.^7^ Low-income countries are increasingly either implementing essential health packages for disease treatment free of charge or providing patients – or their families – with conditional cash transfers for selected health services. Such interventions may often use up a large share of a country’s public health subsidies.^8^

Nepal is a low-income country. In 2011its gross domestic product was 620 United States dollars (US$) per capita.^9^ Since 2006, certain health care services – including the drugs on a national essential drugs list – have been available free of charge at publicly funded district hospitals, health posts, sub-health posts and primary health-care centres.^10^ A Safe Delivery Incentive Programme was implemented throughout Nepal in 2005. This programme has provided pregnant women with cash incentives to encourage institutional delivery and, since 2009, it has also made deliveries free of charge at government facilities and some private facilities.^11^ The Nepalese government subsidizes the treatment of cancers, heart disease, kidney disease and other severe diseases up to a maximum of 50 000 Nepali rupees per patient – just over US$ 500 at the mean exchange rate for 2014.^10^ Although voluntary community-based health insurance schemes are being piloted in six districts of Nepal, their coverage remains sporadic and there is no other publicly-run health insurance scheme in the country.^10^

Despite the treatment subsidies and pilot insurance schemes in Nepal, the incidence and main causes of catastrophic household expenditure on health have not been investigated in detail in the country. It remains unclear if the existing public subsidies that target specific diseases are providing reasonable financial protection to the general population. There have only been a few attempts to determine the effect of disease-specific medical costs on household economic status in southern Asia^5^^,^^12^^,^^13^ or to determine which illnesses have the most impact on household expenditure.^14^^–^^17^ We therefore estimated the incidence of – and determined the illnesses that were most commonly associated with – catastrophic household expenditure on health in an urban area of Nepal.

## Methods

### Study design

We used a multivariate Poisson regression model to analyse self-reported data – on illness and financial expenditure in the previous 30 days – that we collected in a population-based cross-sectional household survey in Kathmandu Valley. The survey covered all five municipalities in Kathmandu Valley: Bhaktapur, Kathmandu, Kirtipur, Lalitpur and Madhyapur-Thimi. We used data from the 2011 national census as the sampling frame and the corresponding census enumeration areas as the primary sampling units. We aimed to sample a total of 2000 households – by multi-stage cluster sampling – between November 2011 and January 2012. We based our choice of sample size on a cluster sampling method, the precision of the estimates required for the study^18^ and an estimate of the prevalence of hypertension in the study area (8%) – assuming that hypertension in those over 20 years of age may represent a major economic burden within the study households.^19^ For the first stage of the sampling, 100 enumeration areas were selected, using systematic sampling with probability proportional to the number of households in each area. For the second stage, a cluster of 20 dwellings was selected in each selected enumeration area. If a selected dwelling contained more than one household, one household in that dwelling was randomly selected. We considered an eligible respondent to be the household head or the most knowledgeable adult in a selected household. To collect data, we used a standardized questionnaire – pre-tested in 100 households in the city of Lalitpur – that included questions on household demographics, education, expenditure and durable goods, self-reported episodes of disease, care-seeking behaviour, total health-related expenditures and inpatient health expenditures, and the coping strategies that household members followed to finance health care (Appendix A; available at http://www.ghp.m.u-tokyo.ac.jp/wp-content/uploads/2014/07/Appendix-A.pdf).

We recorded morbidities that had reportedly occurred in the 30 days before the survey and any chronic conditions that had reportedly continued for more than 3 months in the 12 months before the survey. Each reported illness that had been diagnosed by an allopathic or ayurvedic doctor and the symptoms of any undiagnosed illness were coded according to a disease list that we based on the results of previous studies^12^^,^^20^ and a focus group discussion conducted with health workers in Kathmandu (Appendix A). Whenever possible, interviewers cross-validated a reported diagnosis with the corresponding outpatient card or hospital discharge report. To assess the differences of disease occurrence across economic quintiles we conducted χ^2^ tests.

### Expenditure

The out-of-pocket expenditure on health of each study household – over the 30 days before the survey – was estimated by asking the respondents how much their households had spent, separately, on consultation or diagnosis fees, drugs, other medical supplies and hospitalization costs. The interviewers also posed separate questions on the costs of traditional healers, homeopathic treatments, ayurvedic treatments and home remedies. We also asked each respondent to give a single aggregated estimate of their household’s total expenditure on health in the previous 30 days to see if – as in previous studies^21^^,^^22^ – this estimate fell substantially below the sum of the respondent’s corresponding separate estimates of expenditure on several aspects of health care – i.e. the disaggregated estimate. We used Wilcoxon rank sum test to compare the respondents’ aggregated and disaggregated estimates. Total household expenditure was estimated from the reported consumption, in the 30 days before the survey, of purchased and home-produced goods, including foods, non-foods, housing and durable goods. This estimated expenditure and an adult-equivalent score – based on the number and ages of the members of the household – for each household were then used to identify the economic quintile to which each study household belonged.^23^ Quintiles 1 and 5 represented the poorest and wealthiest households, respectively.

### Comorbidity costs

Some of our study subjects had experienced concurrent episodes of two or more illnesses that were treated concurrently. Such subjects were generally only able to report the total costs of health care for the comorbidities. In these circumstances, we used a regression-based approach – similar to that used by Trogdon et al.^24^ – to allocate a proportion of the jointly reported costs to each illness. More details of such cost allocation are available in Appendix A.

### Catastrophic health expenditure

If, in the 30 days before the survey, a study household had spent more than 10% of its total expenditure on health care, that household was considered to have experienced catastrophic health expenditure in that period.^2^^,^^4^ For the study households in general and for each economic quintile of the study households, we assessed the impact on household economic welfare of out-of-pocket spending on each of the 10 types of illness that were most commonly reported. We used the concentration index^25^ to see if the percentage of households that experienced catastrophic health expenditure was unequally distributed across the five economic quintiles. Concentration indexes with 95% confidence intervals (CI) and their associated *P*-values were calculated using bootstrapping with 100 iterations and the delta method.^26^^–^^28^ The concentration index can range between −1 and +1. In our study, indexes well below and well above zero would indicate that catastrophic expenditure is concentrated among the relatively poor and relatively wealthy households, respectively. We measured the intensity of expenditure burden using catastrophic overshoot, i.e. the average of payments surpassing the catastrophic threshold across all households, expressed as the proportion of additional payments above 10% of the total household consumption and averaged by the total number of households. A concentration index significantly below zero indicates a greater overshoot among the poor. We also report the mean positive overshoot, i.e. the share of additional payments above 10% of the total household consumption, averaged by the number of households with catastrophic expenditure.

### Analysis of risk factors

We used a Poisson regression model to predict the incidence of catastrophic health expenditure among households affected by a particular illness. We stratified the model by household economic quintile to assess the relative risk – of catastrophic household expenditure – posed by each of the commonly reported illnesses in each quintile. The variables included in the model were: whether there was a history of hospitalization in the previous 30 days; the number of people in the household; whether the household had used a health-care provider in the previous 30 days and, if so, whether the provider or providers used by the household in the previous 30 days were public, private or both public and private; the age of the household head; whether the household head had primary or lower, secondary or higher education; the number of children aged less than five years in the household; the number of people aged over 65 years in the household; and whether, in the previous 30 days, a household member had reportedly suffered more than one episode of the 10 most commonly reported illnesses. We adjusted all analyses for the sampling structure of the survey. The results are reported as rate ratio (RR) and 95% CI. All the analyses were performed using Stata version 12.1 (StataCorp. LP, College Station, United States of America).

### Ethical approval

Ethical approval was given by the Ethics Committee of the University of Tokyo and – under registration number 49/2011 – by the Nepal Health Research Council. Written informed consent was obtained from the participating respondents before they were interviewed.

## Results

### Morbidity, provider choices and costs

Some details of the study households are shown in Table 1. As no consenting respondents could be found in three households, data were collected from 1997 (99.8%) of the 2000 selected households. The 10 illnesses that were most commonly reported as occurring among members of the study households – in the 30 days before interview – are shown in Table 2. Cases of common cold and concurrent cough and fever were grouped as cold/cough/fever, since many household members reportedly suffered these complaints simultaneously. Hypertension among household members aged more than 20 years appeared to be positively correlated with household expenditure (Table 2). In the 30 days before interview, members of the study households who needed health care had mostly used just private providers or a combination of private providers with other types of facilities (Appendix A). When comparing the respondents’ aggregated and disaggregated estimates of their households’ out-of-pocket spending on health using a nonparametric test, we found little difference between the two types of estimate (*z* = 0.102, *P* = 0.92).The disaggregated estimates indicated that households in the richest economic quintile spent a considerably smaller share of their total expenditure on health (6.9%) than the other households (range: 8.3% in quintile 3 to 14.8% in quintile 2; Table 3).

**Table 1:** Characteristics of the study households, Nepal, 2011–2012

Household characteristic	Value^a^
**Mean no. of household members (95% CI)**	4.4 (4.3 to 4.5)
**Proportion of household members (95% CI) (*n* = 9177)**	
Aged < 5 years	0.09 (0.07 to 0.10)
Aged > 65 years	0.05 (0.04 to 0.06)
**Mean age of household head, years (95% CI) (*n* = 1997)**	46.1 (44.7 to 47.5)
**No. (%) of household heads (*n* = 1997)**	
Male	1653 (84.2)
Female	344 (15.9)
With no education	532 (24.0)
Educated only to primary level	259 (13.4)
Educated only to secondary level	575 (31.6)
With higher education	631 (31.0)
Owning home	1148 (46.0)
Renting	794 (50.0)
In home provided free of charge	14 (0.6)
Squatting/occupying land illegally	40 (3.4)
Living in other types of dwelling	1 (0.1)

CI: confidence interval.

^a^ Adjusted for sample weights.

**Table 2:** Illnesses most commonly reported as occurring in the previous 30 days, by economic quintile,^a^ Nepal, 2011–2012

Illness	% of households (95% CI)						Difference between groups, *P*^b^
	All (*n* = 1997)	Quintile 1 (*n* = 371)	Quintile 2 (*n* = 359)	Quintile 3 (*n* = 401)	Quintile 4 (*n* = 415)	Quintile 5 (*n* = 451)	
**All household members**							
Cold/cough/fever	12.8 (11.2 to 14.4)	12.9 (10.1 to 15.7)	11.6 (9.4 to 13.8)	13.7 (10.3 to 17.0)	13.2 (9.9 to 16.4)	12.6 (10.5 to 14.8)	0.811
Gastritis/peptic ulcer	3.6 (2.8 to 4.3)	5.5 (3.3 to 7.6)	2.8 (1.8 to 3.7)	3.8 (2.4 to 5.2)	3.4 (2.3 to 4.5)	2.4 (1.5 to 3.2)	0.008
Arthritis	2.9 (2.3 to 3.5)	2.4 (1.6 to 3.2)	4.8 (3.1 to –6.6)	2.7 (1.9 to 3.5)	2.2 (1.4 to 3.0)	2.1 (1.3 to 3.0)	< 0.001
Asthma	1.1 (0.9 to 1.4)	1.3 (0.6 to 2.0)	0.9 (0.3 to 1.5)	1.0 (0.5 to 1.4)	1.5 (0.8 to 2.1)	1.0 (0.6 to 1.5)	0.526
Migraine/headache	0.9 (0.6 to 1.2)	1.3 (0.5 to 2.1)	0.5 (0.2 to 0.8)	1.2 (0.4 to 2.0)	0.9 (0.1 to 2.6)	0.6 (0.2 to 1.1)	0.336
Injury	0.7 (0.5 to 1.0)	1.1 (0.5 to 1.7)	0.4 (0.0 to 0.8)	0.6 (0.2 to 1.0)	0.7 (0.3 to 1.0)	1.0 (0.4 to 1.7)	0.202
Heart disease	0.6 (0.4 to 0.8)	0.8 (0.3 to 1.2)	0.3 (0.1 to 0.6)	0.6 (0.2 to 1.0)	0.5 (0.1 to 0.8)	1.0 (0.5 to 1.4)	0.132
**Household members aged > 20 years**							
Diabetes	3.7 (3.1 to 4.3)	2.3 (1.0 to 3.6)	3.1 (1.7 to 4.5)	3.8 (2.7 to 4.8)	3.8 (2.6 to 5.1)	5.3 (3.8 to 6.8)	0.045
Hypertension	10.5 (9.2 to 11.7)	5.8 (3.9 to 7.8)	9.4 (7.0 to 11.9)	11.9 (9.4 to 14.3)	11.4 (9.1 to 13.7)	13.4 (10.9 to 15.9)	< 0.001
Hyperuricaemia	0.7 (0.3 to 1.1)	0.3 (0.0 to 0.5)	1.6 (0.2 to 3.1)	1.1 (0.0 to 2.3)	1.3 (0.3 to 2.3)	1.0 (0.2 to 1.9)	0.291

CI: confidence interval.

^a^ Quintile 1 represents the poorest households and quintile 5 represents the wealthiest households.

^b^ Calculated using *χ^2^* tests.

**Table 3:** Household out-of-pocket spending on health care in the previous 30 days, by economic quintile,^a^ Nepal, 2011–2012

Expenditure	Households that reported expenditure on health					
	All (*n* = 1 517)	Quintile 1 (*n* = 270)	Quintile 2 (*n* = 275)	Quintile 3 (*n* = 301)	Quintile 4 (*n* = 324)	Quintile 5 (*n* = 347)
**Costs per household, Nepalese rupees (SE)^b^**						
Outpatient	1 999 (202)	1 564 (266)	2 123 (664)	1 559 (149)	2 037 (242)	2 722 (514)
Inpatient	39 657 (6 310)	25 200 (12 437)	51 147 (20 377)	26 059 (8 153)	34 578 (7 170)	50 044 (8 104)
Ayurvedic	861 (138)	301 (55)	907 (251)	828 (131)	759 (460)	1 268 (340)
Other traditional medicine or healers	335 (100)	263 (117)	239 (80)	346 (130)	512 (336)	319 (117)
Transportation and other costs	471 (74)	31 (8)	143 (53)	98 (28)	90 (36)	69 (26)
**Proportion of total household expenditure represented by out-of-pocket spending on health care, % (SE)**	10.1 (1.26)	10.7 (1.55)	14.8 (3.80)	8.3 (1.81)	10.3 (3.24)	6.9 (1.48)

SE: standard error; US$: United States dollars.

^a^ Quintile 1 represents the poorest households and quintile 5 represents the wealthiest households.

^b^ The average conversion rate during the study was 1 Nepalese rupee to US$ 0.012.

### Catastrophic health spending

#### Incidence and intensity

According to the respondents, 13.8% of the study households had experienced catastrophic expenditure on health in the 30 days before interview (Table 4). Such expenditure was most frequently associated with episodes of hypertension, followed – in descending order of frequency – by cold/cough/fever, diabetes and asthma (Table 4). Catastrophic expenditure associated with certain illnesses – such as migraine/headache (concentration index: −0.879; *P* < 0.001) – appeared to be concentrated among the relatively poor households. When we investigated the level by which out-of-pocket treatment costs for each of the commonly reported illnesses exceeded the threshold for catastrophic expenditure, we found that the treatment costs for cold/cough/fever (concentration index: −0.392; *P* < 0.001) and migraine/headache (concentration index: −0.901; *P* < 0.001) appeared to exceed those that the poorer households could bear (Table 5).

**Table 4:** Distribution of catastrophic health expenditure in previous 30 days, divided by major illness, Nepal, 2011–2012

Illness	Catastrophic expenditure			Catastrophic overshoot^a^		Mean positive overshoot (%)^b^
	% of study households (*n* = 1997)^c^	Concentration index (95% CI)		%^c^	Concentration index (95% CI)	
Any	13.8	−0.126 (−0.184 to −0.069)		4.6	−0.045 (−0.195 to 0.105)	33.2
Hypertension	1.3	−0.206 (−0.417 to 0.004)		0.1	−0.224 (−0.462 to 0.116)	10.7
Cold/cough/fever	1.2	−0.262 (−0.459 to −0.066)		0.1	−0.392 (−0.539 to −0.245)	6.8
Diabetes	1.1	−0.099 (−0.304 to 0.107)		0.1	−0.250 (−0.617 to 0.118)	10.2
Asthma	1.0	−0.185 (−0.389 to 0.018)		0.1	0.008 (−0.536 to 0.552)	12.3
Gastritis/peptic ulcer	0.9	−0.111 (−0.447 to 0.225)		0.2	0.364 (−0.111 to 0.839)	17.9
Injury	0.8	−0.033 (−0.328 to 0.261)		0.4	0.011 (−0.479 to 0.501)	49.3
Arthritis	0.7	−0.233 (−0.467 to 0.014)		0.3	−0.395 (−0.830 to 0.041)	41.2
Heart disease	0.5	−0.247 (−0.497 to 0.002)		0.0	−0.194 (−0.511 to 0.122)	8.3
Migraine/headache	0.2	−0.879 (−0.957 to −0.801)		0.0	−0.901 (−0.981 to −0.821)	4.8
Hyperuricaemia	0.2	0.426 (0.379 to 0.473)		0.0	0.426 (0.379 to 0.473)	5.0

CI: confidence interval.

^a^ The mean value by which household out-of-pocket expenditure on the illness – as a percentage of total household expenditure – exceeded the 10% threshold used to define catastrophic household expenditure.

^b^ The mean level by which out-of-pocket expenditure on the illness, by a household reporting catastrophic health expenditure, exceeded the 10% threshold used to define catastrophic household expenditure.

^c^ Adjusted for sampling weight.

**Table 5:** Illness and the risk of catastrophic health expenditure in the previous 30 days, by economic quintile,^a^ Nepal, 2011–2012

Illness**^b^**	Rate ratio (95% CI)				
	Quintile 1	Quintile 2	Quintile 3	Quintile 4	Quintile 5
Diabetes	2.37 (1.16 to 4.83)	2.13 (1.03 to 4.41)	2.85 (1.67 to 4.84)	1.14 (0.61 to 2.13)	1.04 (0.45 to 2.39)
Heart disease	2.24 (1.29 to 3.88)	0.76 (0.26 to 2.27)	1.19 (0.50 to 2.85)	2.17 (0.74 to 6.43)	2.36 (0.83 to 6.71)
Asthma	2.09 (1.28 to 3.42)	1.62 (0.73 to 3.59)	1.94 (1.12 to 3.36)	4.26 (1.89 to 9.61)	1.39 (0.40 to 4.82)
Arthritis	1.72 (0.82 to 3.63)	2.21 (1.24 to 3.94)	1.29 (0.67 to 2.48)	2.32 (1.14 to 4.70)	1.91 (0.75 to 4.88)
Hypertension	1.66 (0.87 to 3.15)	3.26 (1.21 to 8.81)	1.47 (0.81 to 2.67)	1.52 (0.92 to 2.51)	1.62 (0.69 to 3.81)
Migraine/headache	1.64 (0.74 to 3.68)	4.35 (1.71 to 11.04)	1.96 (0.58 to 6.60)	2.29 (0.93 to 5.62)	NA^c^
Gastritis	1.55 (0.76 to 3.17)	1.29 (0.63 to 2.66)	1.32 (0.73 to 2.38)	1.45 (0.77 to 2.74)	2.09 (0.86 to 5.06)
Cold/cough/fever	1.25 (0.57 to 2.73)	2.20 (1.10 to 4.40)	0.85 (0.47 to 1.52)	0.91 (0.43 to 1.94)	0.87 (0.40 to 1.87)
Injury	1.19 (0.35 to 4.03)	3.57 (1.41 to 9.05)	2.58 (1.14 to 5.81)	2.59 (1.32 to 5.09)	3.47 (1.42 to 8.49)
Hyperuricaemia	0.91 (0.38 to 2.16)	1.24 (0.40 to 3.84)	0.11 (0.01 to 0.97)	3.15 (1.65 to 6.00)	1.74 (0.37 to 8.26)

CI: confidence interval; NA: not applicable.

^a^ Quintile 1 represents the poorest households and quintile 5 represents the wealthiest households.

^b^ For each illness, we compared households that had experienced at least one episode with households that had experienced no episodes.

^c^ No episodes of migraine/headache were reported in households in quintile 5.

#### Determinants

The risk of catastrophic spending on health – in the 30 days before interview – varied by the type of illness that affected the household and the economic quintile to which the household belonged (Table 5). For example, in households belonging to the poorest quintile, one or more episodes of diabetes (rate ratio, RR: 2.37; 95% CI: 1.16–4.83), asthma (RR: 2.09; 95% CI: 1.28–3.42) or heart disease (RR: 2.24; 95% CI: 1.29–3.88) were associated with a significantly increased risk of catastrophic expenditure. The occurrence of at least one episode of diabetes increased the risk of catastrophic spending by households in quintiles 2 (RR: 2.13; 95% CI: 1.03–4.41) and 3 (RR: 2.85; 95% CI: 1.67–4.84) but did not significantly increase the risk of such spending by the wealthier households. Injury was associated with an elevated risk of catastrophic spending from the second to the fifth quintile (Table 5).

## Discussion

This study provides evidence relating illnesses to catastrophic out-of-pocket expenditure on health care. More than one in every seven of the households that we investigated in urban areas of Kathmandu Valley reported catastrophic expenditure on health in the previous 30 days. In an earlier nationwide study, using the same definition, the corresponding proportion was only 5.9%.^4^ However, our study focused on urban areas of Nepal, where health facilities are used more frequently than in rural areas.

After adjusting for confounders, we found that major noncommunicable diseases – such as diabetes, asthma and heart disease – were often associated with catastrophic spending in the poorest households. We also found that injury significantly increased the risk of catastrophic expenditure, irrespective of the household’s economic status. A strong relationship between catastrophic expenditure and diabetes was also reported in a review of data from 35 low- and middle-income countries.^14^ In a study in Viet Nam, the households of 27.5% of inpatients receiving treatment for injury had been faced with catastrophic expenditure.^29^

In Nepal there is scope for reducing the economic burden caused by noncommunicable diseases such as diabetes and heart disease. The control and management of the associated risk factors need to be improved, to prevent the onset of the diseases and any further complications. The Islamic Republic of Iran has successfully employed programmes of primary health care, targeted training of health workers and clear guidelines to improve diabetes screening and diagnosis at an early stage.^30^ The regulation of tobacco and alcohol can also reduce the risks of several noncommunicable diseases. The government of Nepal banned tobacco and alcohol advertisements in 1996 and has taxed tobacco and alcohol products for many years. The raising of tobacco prices has been found to be an effective way of reducing tobacco consumption, especially among manual labourers and other low-income groups.^31^ Such interventions can reduce the incidence of some noncommunicable diseases.^32^

It was not surprising to see injuries among the major causes of catastrophic household expenditure in Kathmandu Valley. Although drink-driving is banned in Nepal and the traffic police conduct regular breath tests among drivers in cities, road traffic accidents remain a major cause of injuries requiring treatment in Nepal – as in south-eastern Asia.^33^ In the absence of any general health insurance scheme, serious injury is likely to be associated with unexpected and large household expenditures. The government of Nepal should consider intensifying programmes for the prevention of traffic accidents and injuries in urban municipalities, through road and workplace safety measures such as speed limits and traffic signals.^34^

As a policy priority – for the prevention of health-care-related financial catastrophe in the urban households of Nepal – some form of broad-based risk pooling needs to be encouraged.^6^^,^^35^^,^^36^ The introduction of such a financial protection mechanism may be challenging in Nepal, and with limited fiscal space, a rapid increase in Nepal’s national health expenditure seems unlikely, at least in the short-term.^37^ However, a phased introduction of health insurance or other forms of financial protection may be feasible.^7^^,^^38^

This study has several limitations. First, it was conducted between November 2011 and January 2012 – i.e. in mid-winter. The timing of the survey may well have influenced the recorded prevalence of communicable diseases such as colds, which tend to be more common in winter than in summer. However, in a national survey that took place in 2010–2011 – the Nepal Living Standard Survey – cold/cough/fever was found to be the most prevalent illness throughout the year.^39^ Other studies have also reported a fairly consistent prevalence of diabetes and hypertension in urban Nepal.^40^^,^^41^

The second limitation is that our results are based on self-reported health spending. We assumed that poor households might use coping strategies to minimize their expenditure on health care – e.g. avoiding consultations with physicians, skipping dosages or selecting cheaper medicines. In the treatment of chronic illnesses, non-adherence to prescribed medications is common.^42^^,^^43^ Although respondents were asked whether, to minimize costs, they had ever skipped a dosage, delayed seeking new supplies of medicines or reduced doses, we were not able to quantify how much the respondents may have saved from such cost aversion. Therefore, although, for each of the commonly reported illnesses, we estimated the treatment costs paid by an affected household, these estimates may have been smaller than the full costs of a standard regimen of treatment.

Despite its limitations, this population-based study demonstrates associations between injury and several major diseases and the incidence of catastrophic household expenditure on health care. By identifying the economic burden posed by each type of common illness, it should be possible to prioritize health interventions that are most likely to protect households from impoverishment – even in resource-limited settings.

In Nepal, there is an urgent need to initiate programmes for the control and management of the diseases associated with catastrophic household spending and the prevention of road traffic and other injuries. A phased introduction of health insurance, initially designed to cover or subsidize the costs of care for diabetes and heart disease, should be considered in Nepal. The national government needs to take extra measures to protect the poorest in its population from financial catastrophe.
